# CCAAT/enhancer-binding protein alpha (*CEBPA*) gene haploinsufficiency does not alter hematopoiesis or induce leukemia in *Lck-CALM/AF10* transgenic mice

**DOI:** 10.1590/1414-431X20198424

**Published:** 2019-05-27

**Authors:** A.P. Lange, L.Y. Almeida, C.L. Araújo Silva, P.S. Scheucher, F. Chahud, A. Krause, S.K. Bohlander, E.M. Rego

**Affiliations:** 1 Divisão de Hematologia, Departamento de Clínica Médica, Faculdade de Medicina de Ribeirão Preto, Universidade de São Paulo, Ribeirão Preto, SP, Brasil; 2 Centro de Terapia Celular, Faculdade de Medicina de Ribeirão Preto, Universidade de São Paulo, Ribeirão Preto, SP, Brasil; 3 Departamento de Patologia e Medicina Legal, Faculdade de Medicina de Ribeirão Preto, Universidade de São Paulo, Ribeirão Preto, SP, Brasil; 4 Laboratório de Análises Clínicas Veterinárias, Universidade Federal de Santa Maria, Santa Maria, RS, Brasil; 5 Leukaemia & Blood Cancer Research Unit, Department of Molecular Medicine and Pathology, The University of Auckland, Auckland, New Zealand; 6 Divisão de Hematologia, LIM31, Faculdade de Medicina, Universidade de São Paulo, São Paulo, SP, Brasil

**Keywords:** Leukemia, CEBPA, CALM/AF10, Transgenic mice

## Abstract

Although rare, *CALM/AF10* is a chromosomal rearrangement found in immature T-cell acute lymphoblastic leukemia (T-ALL), acute myeloid leukemia, and mixed phenotype acute leukemia of T/myeloid lineages with poor prognosis. Moreover, this translocation is detected in 50% of T-ALL patients with gamma/delta T cell receptor rearrangement, frequently associated with low expression of transcription factor CCAAT/enhancer-binding protein alpha (*CEBPA*). However, the relevance of *CEBPA* low expression for *CALM/AF10* leukemogenesis has not yet been evaluated. We generated double mutant mice, which express the *Lck-CALM/AF10* fusion gene and are haploinsufficient for the *Cebpa* gene. To characterize the hematopoiesis, we quantified hematopoietic stem cells, myeloid progenitor cells, megakaryocyte-erythrocyte progenitor cells, common myeloid progenitor cells, and granulocyte-macrophage progenitor cells. No significant difference was detected in any of the progenitor subsets. Finally, we tested if *Cebpa* haploinsufficiency would lead to the expansion of Mac-1^+^/B220^+^/c-Kit^+^ cells proposed as the *CALM/AF10* leukemic progenitor. Less than 1% of bone marrow cells expressed Mac-1, B220, and c-Kit with no significant difference between groups. Our results showed that the reduction of *Cebpa* gene expression in *Lck-CALM/AF10* mice did not affect their hematopoiesis or induce leukemia. Our data corroborated previous studies suggesting that the *CALM/AF10* leukemia-initiating cells are early progenitors with lymphoid/myeloid differentiating potential.

## Introduction

The *CALM/AF10* fusion gene derived from the t(10;11)(p13;q14) chromosomal translocation can be found in T-cell acute lymphoblastic leukemia (T-ALL), acute myeloid leukemia (AML) and mixed phenotype acute leukemia (MPAL) of T/myeloid lineages (1–5). The leukemogenic activity of *CALM/AF10* was demonstrated in several murine models. Mice transplanted with murine bone marrow (BM) cells expressing *CALM/AF10* fusion transcripts developed an aggressive form of biphenotypic leukemia (6). Accordingly, Caudell et al. (7) generated transgenic mice (TM) expressing *CALM/AF10* under the control of the *vav* promoter (a pan-hematopoietic vector) and approximately 40-50% of TM developed leukemia at the median age of 12 months. The leukemic blasts isolated from the BM and spleen of *vav*-*CALM/AF10* TM co-expressed Mac-1, B220, and CD24 with variable expression of CD117 in the absence of other B-cell markers, thus resembling human MPAL with involvement of B and myeloid lineages. However, Abdelali et al. (8) screened 665 patients with T-ALL and identified *CALM-AF10* in 30/431 (7%) patients aged 16 years and over, and in 15/234 (6%) of those aged up to 15 years of age.

Aberrant expression of T-cell lineage markers on AML blasts has been associated with mutations or epigenetic changes of the *CEBPA* gene or its promoter (9). *CEBPA* encodes for a transcription factor of crucial relevance for granulocytic differentiation (10). In addition to mutations in the *CEBPA* gene, which were detected in approximately in 6−15% of *de novo* AML and in 15−18% of AML with normal karyotypes (11), silencing of *CEBPA* expression due to promoter hypermethylation was found in a small subset of AML patients with myeloid/T-lymphoid features (12). In the study by Terriou et al. (13), half of the *CEBPA* methylated T-ALL cases were *CALM/AF10* positive (5/10), suggesting that the search of *CALM/AF10* in *CEBPA* methylated AML could help distinguish between the immature T-ALL and myeloperoxidase negative AML. Based on the existing data showing the aberrantly low expression of the *CEBPA* gene in patients with acute leukemia harboring the *CALM/AF10* fusion gene, we decided to evaluate *in vivo* if haploinsufficiency of *CEBPA* predisposes *CALM/AF10* ^+^ hematopoietic cells to develop leukemia.

## Material and Methods

### Generation of double mutant mice

Mice haploinsufficient for the *Cebpa* gene (*Cebpa* ^+/-^) were kindly provided by Prof. Daniel G. Tenen (National University of Singapore) and were crossed to *Lck*-*CALM/AF10* TM, in which the expression of the fusion gene is under the control of the *lck* regulatory region. The *Lck*-*CALM/AF10* TM were kindly provided by Prof. Stefan K Bohlander (University of Auckland). The phenotype of parental lines has been previously described (14). The offspring was genotyped by conventional polymerase chain reaction (PCR) (Table 1).

**Table 1:** Primers used for mouse genotyping.

Genotyping reaction	Direction	Primer (5′-3′)	Product Size (pb)	Annealing temperature (°C)
PCR CALM/AF10	F	5′-CCAAACTCCCACCTAGCAAGTTAG-3′	1023	57°C, 45 s
	R	5′-GGTGTGTGCAGAGACTTCCTG-3′		35 cycles
PCR Cebpa^(+/-)^	F	5′-GCCTACCGGTGGATGTGGAATGTG-3′	300 and 500	56°C, 1 min
	R	5′-ACTTAGGTGTTGGGGATTTGAGTCTGTG-3′		35 cycles
	F	5′-TCCCCCAGCCGTTAGTGAAGAGTCTC-3′		
PCR beta-actin	F	5′-TCTTGATAGTTCGCCATGGAT-3′	500	64°C, 30 s
	R	5′-GGTCATCTTTTCACGGTTGG-3′		30 cycles

F: forward; R: reverse.

### Serial analysis of hematological counts

Animals aged 3–15 months were monitored once every three months for peripheral blood counts. Leishman-stained blood smears were used for differential leukocyte counts. All animal experiments were performed after the approval of the Animal Care and Use Committee of the Medical School of Ribeirão Preto of the University of São Paulo (Protocol number: 144/2013) in strict accordance with institutional guidelines regulated by the National Council of Animal Experimentation (CONCEA-Brazil). All experimental procedures were performed under isoflurane anesthesia, and all efforts were made to minimize suffering.

### Flow cytometry analysis of BM hematopoietic progenitors and mature cells

Five to seven mice of each group were sacrificed with anesthetic overdose at the age of 15 months and BM single cell suspensions were prepared by crushing femurs, tibiae, and iliac crests with a pestle. The percentage of mature BM cells was identified using murine markers for erythroid precursors (anti-TER-119, clone TER-119), granulocytes (anti-CD11b/Mac-1, clone M1/70), and B (anti-B220, clone RA3-6B2) and T cells (anti-CD3, clone 145-2C11). All antibodies were purchased from BD Pharmingen, USA. To identify the hematopoietic progenitor populations, BM cells were depleted of mature erythroid, myeloid, and lymphoid lineages using the Lineage Cell Detection Cocktail-Biotin, mouse (Miltenyi Biotec, USA). The remaining cells were stained for anti-Ly-6A/Sca-1 (clone D7, Biolegend, USA), anti-CD34 (clone RAM34, eBioscience, USA), CD16/32 (clone 2.4G2, Biolegend), anti-CD117/c-kit (clone 2B8, Biolegend), anti-B220 (clone RA3-6B2, BD Pharmingen), anti-CD3 (clone 145-2C11, BD Pharmingen), anti-Cd11b/Mac-1 (clone M1/70, BD Pharmingen), and anti-TER119 (clone TER-119, BD Pharmingen). The following progenitor populations were quantified: HSC (hematopoietic stem cells, Lin^-^ Sca-1^+^ c-Kit^+^), MP (myeloid progenitor cells, Lin^-^ Sca-1^-^ c-Kit^+^), MEP (megakaryocyte-erythrocyte progenitor cells, Lin^-^ Sca-1^-^ c-Kit^+^ Fcƴ^lo^ CD34^-^), CMP (common myeloid progenitor cells, Lin^-^ Sca-1^-^ c-Kit^+^ Fcƴ^lo^ CD34^+^), and GMP (granulocyte-macrophage progenitor cells, Lin^-^ Sca-1^-^ c-Kit^+^ Fcƴ^hi^ CD34^+^). Samples were analyzed on a JSAN cell sorter (Bay biosciences, Japan) after exclusion of dead cells using DAPI staining. Data were analyzed with FlowJo software (TreeStar, USA).

### Histopathological analysis

Vertebrae and spleens from animals used for flow cytometry analysis were first fixed in PBS with 4% paraformaldehyde and processed for paraffin-embedded sectioning at 5 μm in thickness, and then stained with hematoxylin & eosin.

## Results

**Figure 1:**
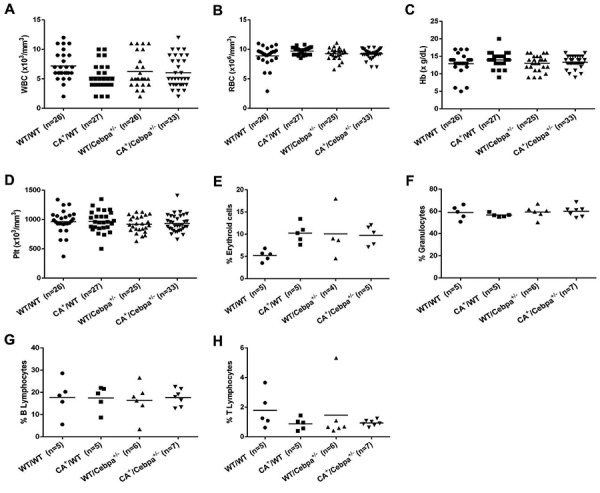
Hematological parameters and immunophenotypic characterization of bone marrow cells of adult mice (9 to 15 months). **A**, WBC: leukocyte counts. **B**, RBC: red blood cell counts. **C**, Hb: hemoglobin concentrations. **D**, Plt: platelet counts. **E**, Percentage of erythroid cells. **F**, Percentage of granulocytes. **G**, Percentage of B lymphocytes. **H**, Percentage of T lymphocytes. Each dot represents an individual mouse and lines indicate the median. *WT/WT*: wild-type (control); *CA* ^+^/*WT*: *CALM/AF10*; *WT/Cebpa* ^+/-^: *Cebpa* ^+/-^; *CA* ^+^/*Cebpa* ^+/-^: double-mutant mice. No significant difference was found between groups. Statistical analysis was performed with the Kruskal-Wallis test followed by Dunn's multiple comparison tests.

After a median follow-up of 15 months, no significant difference in the median values of leukocyte counts, red blood cell counts, hemoglobin concentrations, and platelet counts were detected among the four genotypes. Figure 1A–D shows the main hematological parameters of peripheral blood observed in last sample collection during monitoring, but similar results were found at all time-points. Moreover, there was no change in the percentage of erythroid precursors, granulocytes, and B and T lymphocytes in the BM (Figure 1E–H). Of note, no case of leukemia was detected among 23 mice of the *CA* ^+^/*WT* group and the 33 animals of the *CA* ^+^/*Cebpa* ^+/-^ group. The number of animals was adequate for the objectives and followed the good practice guide for the use of animals in research. Our results were in agreement with Krause (14) who did not detect leukemia in *Lck*-*CALM/AF10* TM.

*CALM/AF10* rearrangements have been detected in patients with acute leukemia of different phenotypes, but are more frequently associated with T-ALL (2 [Bibr B03] [Bibr B04],^5^). Terriou et al. (15) evaluated *CEBPA* promoter methylation in 5 AML and 99 T-ALL cases, with expression of cytoplasmic CD3 and CD7 transcripts, T-cell receptor rearrangement, and *NOTCH1* mutations. Half of the cases with hypermethylated *CEBPA* regulatory regions were T-ALL *CALM/AF10* positive. These findings suggest that the low expression of *CEBPA* caused by epigenetic mechanisms may play a role in *CALM/AF10* leukemogenesis.

In the present study, *CALM/AF10* expression was driven by the *Lck* proximal promoter region. During normal T-cell ontogenesis, *Lck* transcription is regulated by two distinct promoter elements. The proximal promoter is active almost exclusively in thymocytes and becomes inactive later during T-cell maturation and has been used experimentally to drive the expression of the transgene to T-cell lineage (16–17). However, Bell and Bhandoola (18) demonstrated that the earliest thymic progenitors have not only lymphoid but also myeloid potential during hematopoiesis, providing alternative phenotypes for a lineage specification of a potential leukemic transformation driven by an *lck-CALM/AF10* translocation. In our model, *CALM/AF10* expression occurred in hematopoietic progenitors with restricted capacity of differentiation and our results suggest that *CALM/AF10* may not be leukemogenic in this cell compartment. Reinforcing this hypothesis, Caudell et al. (7) generated a murine model in which the expression of *CALM/AF10* was targeted to multipotent progenitors using the *vav* promoter, and reported that approximately 50% of *vav-CALM/AF10* TM developed leukemia. The leukemic blasts showed myeloid differentiation associated with lymphoid features, thus resembling human MPAL. However, the long latency of the disease in this model further argues that additional events are necessary for the development of leukemia.

## Discussion

**Figure 2:**
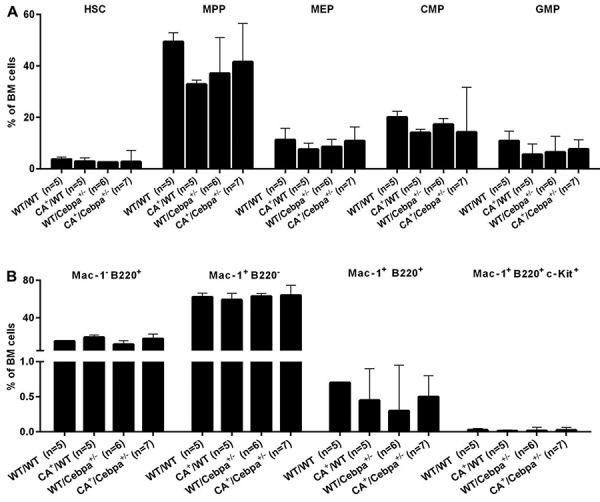
Immunophenotypic characterization of bone marrow (BM) hematopoietic progenitor cells between mice groups. *WT/WT*: wild-type (control); *CA* ^+^/*WT*: *CALM/AF10*; *WT/Cebpa* ^+/-^: *Cebpa* ^+/-^; *CA* ^+^/*Cebpa* ^+/-^: double-mutant mice. **A**, Frequencies of HSC (hematopoietic stem cells, Lin^-^ Sca1^+^ c-Kit^+^), MP (myeloid progenitor cells, Lin^-^ Sca1^-^ c-Kit^+^), MEP (megakaryocyte-erythrocyte progenitor cells, Lin^-^ Sca^-^ c-Kit^+^ Fcƴ^lo^ CD34^-^), CMP (common myeloid progenitor cells, Lin^-^ Sca^-^ c-Kit^+^ Fcƴ^lo^ CD34^+^), and GMP (granulocyte-macrophage progenitor cells, Lin^-^ Sca^-^ c-Kit^+^ Fcƴ^hi^ CD34^+^) of *WT/WT*, *CA* ^+^/*WT*, *WT/Cebpa* ^+/-^, and *CA* ^+^/*Cebpa* ^+/-^ mice. Data are reported as means and standard deviations. **B**, Bone marrow hematopoietic progenitors with features of leukemic stem cells Mac-1^-^B220^+^ cells, Mac-1^+^B220^-^ cells, Mac-1^+^B220^+^ cells, and Mac-1^+^B220^+^ c-Kit^+^ cells. No significant difference was observed. Statistical analysis was performed with Kruskal-Wallis test followed by Dunn's multiple comparison tests.

**Figure 3:**
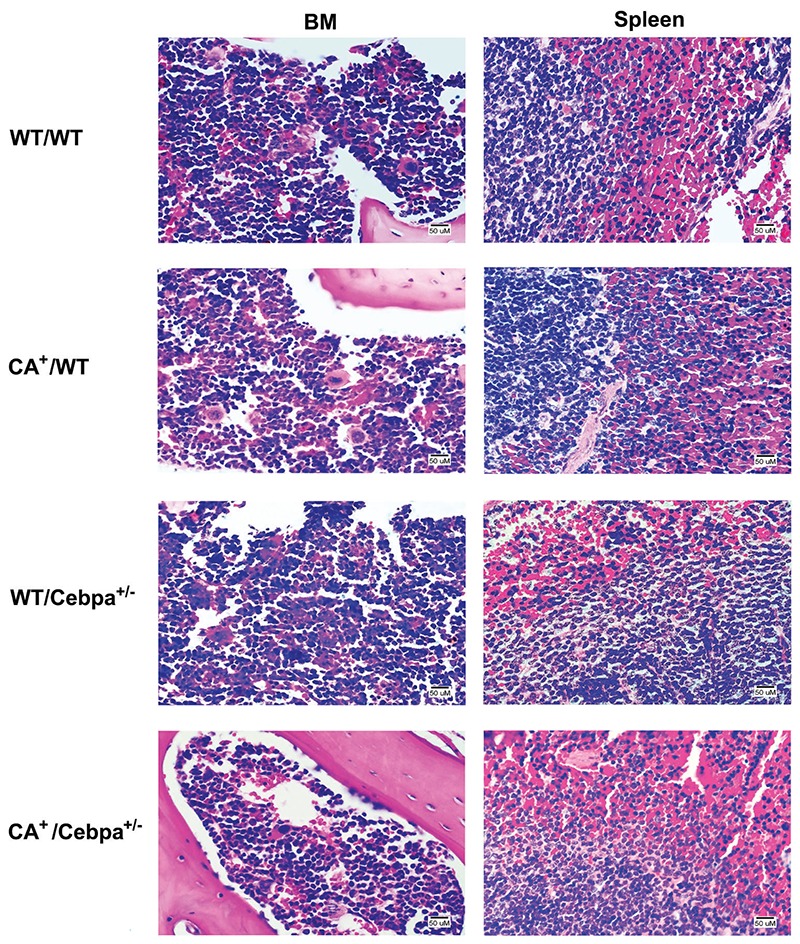
Histopathological analysis of the bone marrow (BM) and spleen from *WT/WT*: wild-type (control); *CA* ^+^/*WT*: *CALM/AF10*; *WT/Cebpa* ^+/-^: *Cebpa* ^+/-^; *CA* ^+^/*Cebpa* ^+/-^: double-mutant mice. The normal architecture of the bone marrow and spleen was preserved in *CA* ^+^/*WT*, *WT/Cebpa* ^+/-^, and *CA* ^+^/*Cebpa* ^+/-^ mice and showed no changes in the cellularity pattern compared to control mice. Hematoxylin and eosin stain. Bar: 50 µm.

In order to fully characterize the hematopoiesis in *WT/WT*, *CA* ^+^/*WT*, *WT/Cebpa* ^+/-^, *CA* ^+^/*Cebpa* ^+/-^ mice, we examined whether there were genotype-specific differences in BM HSC and myeloid progenitors (MP, MEP, CMP, and GMP). Frequencies of HSC, MP, MEP, CMP, and GMP did not differ between groups (Figure 2A). In addition, BM and spleen did not show differences between the groups on histopathological analysis (Figure 3).

Decreased expression of *CEBPA* plays a role in acute promyelocytic leukemia (APL) leukemogenesis. Guibal et al. reported that *Cebpa* ^+/-^ mice did not develop leukemia nor present abnormal cell counts, but double mutants human cathepsin G (hCG)-*PML/RARA Cebpa* ^+/-^ developed APL at a higher frequency, decreased latency, and had a significantly shorter survival rate than hCG-*PML/RARA* TM. Furthermore, the APL-initiating cells showed decreased mRNA and protein expression of *CEBPA* compared with normal promyelocytes (19).

Deshpande et al. (6) showed that, in a transplantation model of murine CALM/AF10^+^leukemia, the myeloid leukemia cells were frequently B220^+^, with clonal B cell receptor (BCR) rearrangement and lacked c-Kit expression, demonstrating that these AML cells had lymphoid characteristics. In contrast, in the study of Caudell et al. (7), only 50% of the AML from *vav-*CALM/AF10 TM were B220^+^ and exhibited clonal BCR rearrangement, with variable expression of c-Kit. Nevertheless, both studies showed that Mac-1^+^/B220^+^ cells lacked specific B‐cell markers. Dutta et al. (20) studied the role of *CALM/AF10* specifically in B cell development and function in Mb1-Cre or CD19-Cre mice and demonstrated that expression of *CALM/AF10* in the B cell compartment did not lead to leukemia. Together, these authors show evidence that the cell of origin of CALM/AF10 AML with lymphoid characteristics is an early progenitor not derived from B-cell lineage. In order to test if there is an expansion of the rare population of hematopoietic progenitor cells (Mac-1^+^/B220^+^) followed or not by the expression of c-Kit in the BM of double mutant mice *CA* ^+^/*Cebpa* ^+/-^, we quantified the frequency of these cells in our model. Less than 1% of BM cells expressed Mac-1, B220, and c-Kit with no significant difference between groups (Figure 2B).

In summary, our results showed that the reduction of *Cebpa* expression did not contribute to leukemogenesis in *Lck*-*CALM/AF10* mice and did not affect hematopoiesis. In future experiments, it will be important to determine if decreased *Cebpa* expression can modify leukemia frequency or promote the expansion of specific cell subsets in *vav-CALM/AF10/Cebpa* ^+/-^ double mutant mice. Nevertheless, our data support the hypothesis that the normal counterpart of the *CALM/AF10* ^+^ leukemia-initiating cells is a hematopoietic stem cell or an early progenitor with myeloid and lymphoid potential.
